# Baseline homeostasis model assessment of insulin resistance associated with fibrosis progression in patients with nonalcoholic fatty liver disease without diabetes: A cohort study

**DOI:** 10.1371/journal.pone.0255535

**Published:** 2021-08-25

**Authors:** Dae-Jeong Koo, Mi Yeon Lee, Inha Jung, Sun Joon Moon, Hyemi Kwon, Se Eun Park, Eun-Jung Rhee, Won-Young Lee

**Affiliations:** 1 Division of Endocrinology and Metabolism, Department of Internal Medicine, Changwon Fatima Hospital, Changwon, Republic of Korea; 2 Division of Biostatistics, Department of R&D Management, Kangbuk Samsung Hospital, Sungkyunkwan University School of Medicine, Seoul, Republic of Korea; 3 Division of Endocrinology and Metabolism, Department of Internal Medicine, Kangbuk Samsung Hospital, Sungkyunkwan University School of Medicine, Seoul, Republic of Korea; Kaohsiung Medical University Chung Ho Memorial Hospital, TAIWAN

## Abstract

**Background and aims:**

Fibrosis progression is the most important prognostic factor, and insulin resistance is one of the main mechanisms associated with fibrosis progression in patients with nonalcoholic fatty liver disease (NAFLD). We evaluate the association between baseline insulin resistance and future fibrosis progression in patients with NAFLD without diabetes.

**Approach and results:**

This retrospective longitudinal study with 8-year follow-up period included 32,606 (men, 83%) participants aged >20 years (average age, 38.0 years) without diabetes at baseline who completed at least two comprehensive health checkups from January 1, 2010 to December 31, 2018. NAFLD was diagnosed based on ultrasonography. The homeostasis model assessment of insulin resistance (HOMA-IR) was used to evaluate baseline insulin resistance. Fibrosis progression was assessed using the aspartate aminotransferase to platelet ratio index (APRI). The advanced liver fibrosis with an APRI value above the intermediate fibrosis probability (≥0.5) developed in a total of 2,897 participants during 136,108 person-years. 114 participants progressed to a high fibrosis probability stage (APRI >1.5) during 141,064 person-years. Using the lowest baseline HOMA-IR quartile group (Q1) as a reference, the multivariate-adjusted hazard ratio (HR) for development of advanced liver fibrosis (APRI ≥0.5) in the highest baseline HOMA-IR quartile group (Q4) was 1.95 (95% confidence interval [CI] 1.74–2.19; Model 4). And the HR for development of advanced liver fibrosis with high fibrosis probability was 1.95 (95% CI 1.10–3.46; Model 4). The positive association was maintained throughout the entire follow-up period. The baseline HOMA-IR model was superior to the baseline body mass index (BMI) model in predicting the progression of fibrosis probability.

**Conclusions:**

In this longitudinal study, we found that the degree of baseline insulin resistance, assessed by HOMA-IR values, was positively associated with future fibrosis progression in patients with NAFLD without diabetes.

## Introduction

Nonalcoholic fatty liver disease (NAFLD) is the most common liver-related disease worldwide and is increasing in proportion to the growing population with obesity and metabolic syndrome [1]. NAFLD can be diagnosed when steatosis of hepatocytes exceeds 5% histologically, and other secondary factors, such as alcohol, should be excluded [2]. The worldwide prevalence of NAFLD is estimated to be approximately 25% [3]. NAFLD is associated with increased mortality due to cardiovascular disease [4] and liver disease itself. The stage and progression of fibrosis are the most important factors in predicting the prognosis of patients with NAFLD [5].

Unfortunately, approximately 20% of patients with NAFLD progress to nonalcoholic steatohepatitis (NASH) and fibrosis, and approximately 20% of patients with NASH progress to cirrhosis [6]. Given that there is currently no established medical treatment for biopsy-proven NASH and liver fibrosis other than lifestyle modification, it is very important to prevent progression in the early stages of NAFLD [2, 7]. From this point of view, finding a simple and reliable metabolic factor capable of predicting the progression of fibrosis in patients with NAFLD is a major concern for clinicians.

NAFLD is regarded as a disease in which metabolic syndrome affects the liver [8] and has been significantly associated with abdominal obesity as well as types 1 and 2 diabetes [9–11]. The core pathophysiology leading to NASH, fibrosis, and cirrhosis, as well as the development of steatosis, is thought to be insulin resistance (IR) [12–14]. The homeostasis model assessment of IR (HOMA-IR) is an easy and useful method of evaluating IR and beta cell function [15, 16]. To date, liver biopsy is the diagnostic gold standard for liver fibrosis in patients with NAFLD that best reflects histological changes in the liver [7]. However, biopsy has limitations such as cost, difficulty in interpretation, and risk of mortality [17]. In particular, it is practically impossible to apply biopsy as a routine in studies based on a large cohort involving a relatively healthy general population. In addition to imaging techniques such as elastography, scoring panels using biomarkers can be applied to predict or diagnose advanced fibrosis in clinical settings [18].

In patients with NAFLD proven by biopsy, regardless of diabetes, the baseline HOMA-IR can be an independent predictor of fibrosis progression [19, 20], and the HOMA-IR at the time of diagnosis was associated with the stage of fibrosis [21–23]. Only 1 study reported the association between baseline HOMA-IR and the risk of fibrosis progression in patients with NAFLD as evaluated using a non-invasive scoring index regardless of the presence of diabetes; however, the main focus of the study was weight change, not HOMA-IR [24]. In this study, we performed a longitudinal study to evaluate the association between baseline HOMA-IR and the incidence of advanced liver fibrosis as evaluated by aspartate aminotransferase to platelet ratio index (APRI), in a large cohort of relatively young and middle-aged Korean adults with NAFLD without diabetes.

## Patients and methods

### Study participants

This was a retrospective cohort study of adults aged >20 years who completed at least 2 comprehensive health checkups at the Total Healthcare Center of Kangbuk Samsung Hospital in Seoul and Suwon, South Korea, from January 1, 2010 to December 31, 2018.

**Fig 1:**
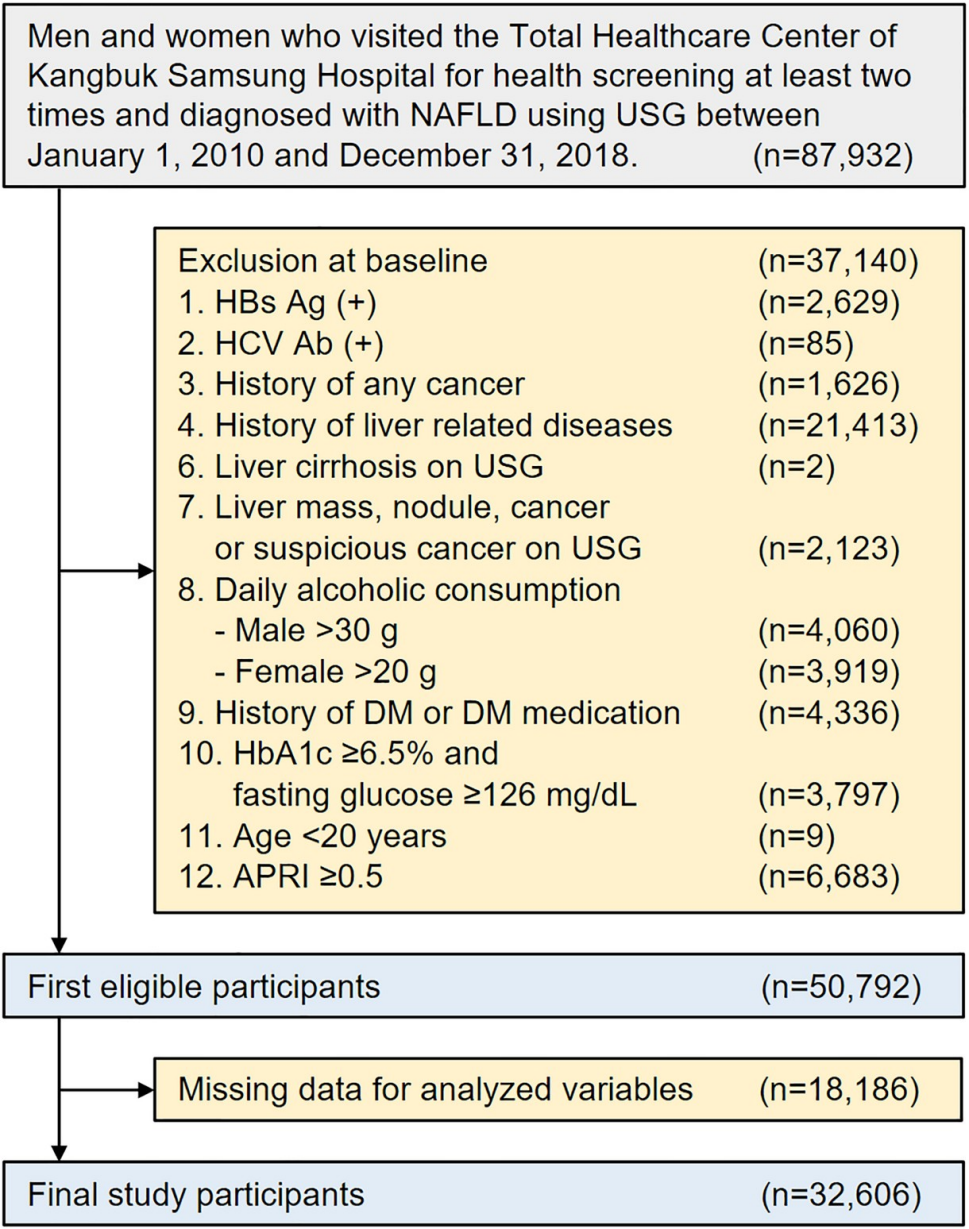
Flow chart of study participants. Abbreviations: NAFLD, nonalcoholic fatty liver disease; USG, ultrasonography; DM, diabetes mellitus; APRI, aspartate aminotransferase-to-platelet ratio index.

Among subjects who visited our center ≥2 times during the observation period, 87,932 participants diagnosed with NAFLD using abdominal ultrasonography (USG) were selected; 37,140 participants were excluded for the following reasons at baseline: positive findings for serologic markers of hepatitis B (n = 2,629) or hepatitis C infection (n = 85); history of cancer (n = 1,626); history of other liver disease including cirrhosis (n = 21,413); liver cirrhosis on abdominal USG (n = 2); liver mass, nodule, cancer, or suspicious cancer lesions on abdominal USG (n = 2,123); daily alcohol intake of ≥30 g/day for men (n = 4,060) or ≥20 g/day for women (n = 3,919); history of diabetes or use of antidiabetic medications (n = 4,336); hemoglobin A1c (HbA1c) levels ≥6.5% and fasting glucose levels ≥126 mg/dL (n = 3,797) [25]; age <20 years (n = 9); aspartate transaminase to platelet ratio index (APRI) ≥0.5 (n = 6,683); and missing data on blood pressure, BMI, waist circumference, HbA1c, high-sensitivity C-reactive protein (hs-CRP), high-density lipoprotein (HDL) cholesterol, low-density lipoprotein (LDL) cholesterol, or triglyceride (n = 18,186). The final sample size for our study was 32,606 (27,089 men, 83.08%) (Fig 1). This study was approved by the Institutional Review Board of Kangbuk Samsung Hospital (KBSMC 202006110001), which waived the requirement for informed consent because we retrospectively accessed data from a de-identified database.

### Anthropometric and laboratory measurements

Medical history, alcohol consumption, smoking status, and exercise status were acquired using a self-administered questionnaire. The questionnaire was based on the 4th National Health and Nutrition Survey [26] and Korean version of the International Physical Activity Questionnaire short form [27]. Alcohol intake was determined based on the average number of drinks per week, and current smoking status was based on the response “yes” or “no.” Regular exercise was defined as moderate- or high-intensity exercise performed >3 times per week. For body weight measurements, the subjects wore a thin gown, and BMI was calculated by dividing body weight (kg) by the squared height (m^2^). Blood pressure (BP) was measured in both arms at an interval of ≥1 min in a sitting position using a standard sphygmomanometer in a stable state after sufficient rest, and the higher of the 2 values was recorded. If the systolic blood pressure (SBP) or diastolic blood pressure (DBP) exceeded 140 mmHg or 90 mmHg, respectively, the measurement was repeated after 5 min, and the results were averaged. All blood samples were collected from patients after an overnight fast of ≥12 h. To assess fasting blood glucose and insulin levels, the hexokinase method (Modular D2400; Hitachi, Tokyo, Japan) and electrochemiluminescence immunoassay method were used (Modular E170; Hitachi), respectively. The enzymatic colorimetric method was used to measure total cholesterol and triglyceride levels. HDL cholesterol levels were measured using a selective inhibition method, and LDL cholesterol levels were measured using a homogeneous enzymatic colorimetric test. An immunoturbidimetric assay (Cobra Integra 800 automatic analyzer; Roche Diagnostics, Basel, Switzerland) was used to measure HbA1c levels. Aspartate transaminase (AST) and alanine aminotransferase levels were measured using Bayer Reagent Packs on an automated chemistry analyzer (Advia 1650 Autoanalyzer; Bayer Diagnostics, Leverkusen, Germany), and a nephelometric assay (BNII nephelometer; Dade Behring, Deerfield, IL, USA) was used to measure hs-CRP levels. To determine IR, we used the HOMA-IR formula: HOMA-IR = (fasting insulin [μIU/mL] × fasting blood glucose [mg/dL] /405) [15].

### Evaluation of NAFLD and liver fibrosis

Fatty liver was examined using abdominal USG (Logic Q700 MR; GE, Milwaukee, WI, USA) and diagnosed based on USG images showing evidence of hepatic steatosis, such as higher echogenicity compared with the kidney or spleen, attenuation of the ultrasound wave, loss of definition of the diaphragm, vessel wall brightness, and poor delineation of the intrahepatic architecture [28, 29]. We defined NAFLD as fatty liver without any other cause of hepatic steatosis [7].

To assess the degree of liver fibrosis in participants with NAFLD, the APRI was applied [30], where APRI = ([AST/upper limit of normal]/platelet [10^9^/L]) × 100. The cutoff values for fibrosis probability estimation based on APRI category were as follows: <0.5, low fibrosis probability; 0.5–1.5, intermediate fibrosis probability; and >1.5, high fibrosis probability; we defined advanced liver fibrosis in NAFLD as when a participant had an APRI value above the intermediate fibrosis probability (≥0.5) [31].

### Statistics

The development of advanced liver fibrosis was the primary endpoint in this study. A parametric proportional hazards model adjusted for multivariate using 95% confidence interval (CI), controlling the interval censored events, was applied to analyze the association between baseline HOMA-IR and the incidence for the development of advanced liver fibrosis [32]. For the estimation of baseline log cumulative hazards, restricted cubic splines were used. Multivariate models have been adjusted using confounding variables associated with NAFLD. To control the effects of new-onset diabetes and HOMA-IR change, we further adjusted for time-varying development of diabetes and HOMA-IR changes during the follow-up period. (Model 4).

We also performed predefined subgroup analyses by age (<45, ≥50 years), sex, BMI (<25, ≥25 kg/m^2^), exercise status (<3 times/week, ≥3 times/week), current alcohol consumption (defined as daily alcohol consumption above the median value: 12 g/day for men and 2 g/day for women), and dyslipidemia (defined as LDL cholesterol ≥130 mg/dL, total cholesterol ≥200 mg/dL, triglyceride ≥150 mg/dL, and HDL <40 mg/dL in men and <50 mg/dL in women; or current use of antidyslipidemic drugs).

To quantitatively evaluate the relative power of baseline HOMA-IR and BMI values in predicting advanced liver fibrosis, the continuous variables were standardized and classified into quartiles, and hazard ratios (HRs) were also calculated. Moreover, the Akaike information criterion (AIC) calculation was performed to compare the probability of the development of advanced liver fibrosis between baseline HOMA-IR and BMI values.

Values in the tables of this study are expressed as means±SDs, numbers (%), or medians (interquartile ranges). All reported 2-tailed *p*-values of <0.05 were considered statistically significant. All statistical analyses were performed using STATA version 16.1 (StataCorp, College Station, TX, USA).

## Results

### General characteristics of study participants

Most of the 32,606 study participants were relatively young men (mean age 38.0 ± SD 7.7 years, male 83%). The mean BMI gradually increased from the quartile group with the lowest baseline HOMA-IR (group Q1) to the group with the highest baseline HOMA-IR (group Q4), and the proportion of obese individuals in group Q4 was more than twice that in group Q1 (80.2% vs. 39.5%). As baseline HOMA-IR quartile increased, the levels of baseline metabolic parameters and lifestyle conditions associated with increased IR or NAFLD progression were increasingly unfavorable except for smoking status (Table 1) [2, 4, 7]. Participants who developed advanced liver fibrosis (APRI ≥0.5) had more adverse levels of baseline metabolic parameters associated with increased IR or NAFLD progression than those who did not (S1 Table).

**Table 1:** Baseline characteristics of participants by HOMA-IR quartile at baseline.

Characteristics	Overall	Baseline HOMA-IR quartile (range)				*p*
		Q1 (0.07–1.13)	Q2 (1.14–1.65)	Q3 (1.66–2.35)	Q4 (2.36–26.15)	
**Number of participants**	32,606	8,109	8,165	8,141	8,191	
**Male, n (%)**	27,089 (83.1)	6,907 (85.2)	6,952 (85.1)	6,877 (84.5)	6,353 (77.6)	<0.001
**Age (years)**	38.0±7.7	39.3±8.0	38.5±7.8	37.6±7.5	36.5±7.3	<0.001
**BMI (kg/m^2)^**	26.0±3.0	24.5±2.3	25.5±2.5	26.2±2.7	27.9±3.4	<0.001
**Obesity^*a*^, n (%)**	19,662 (60.3)	3,204 (39.5)	4,544 (55.7)	5,343 (65.6)	6,571 (80.2)	<0.001
**WC (cm)**	89.1±7.8	85.5±6.5	87.9±6.8	89.8±7.0	93.4±8.5	<0.001
**FBG (mg/dL)**	95.49±9	90.5±7.7	94.4±7.7	96.72±8.0	100.4±9.4	<0.001
**Hemoglobin A1c (%)**	5.6±0.3	5.6±0.3	5.6±0.3	5.6±0.3	5.7±0.3	<0.001
**SBP (mmHg)**	115.8±12.1	113.1±11.7	115.1±11.8	116.2±11.8	118.9±12.4	<0.001
**Antihypertensive medications, n (%)**	1,827 (5.6)	351 (4.3)	412 (5.1)	455 (5.6)	609 (7.4)	<0.001
**Hypertension, n (%)**	3,637 (11.2)	663 (8.2)	811 (9.9)	913 (11.2)	1,250 (15.3)	<0.001
**AST (U/L)**	23 (19–28)	22 (19–27)	22 (19–27)	23 (19–28)	24 (19–30)	<0.001
**ALT (U/L)**	28 (20–40)	24 (18–33)	27 (19–37)	29 (21–41)	34 (23–49)	<0.001
**Platelets (×10^3^/mm^3)^**	255.6±49.8	249.00±47.6	253.5±48.9	255.9±49.1	264.2±52.3	<0.001
**Albumin (g/dL)**	4.7 (4.5–4.9)	4.7 (4.5–4.9)	4.7 (4.5–4.9)	4.7 (4.5–4.9)	4.7 (4.5–4.9)	<0.001
**Total cholesterol (mg/dL)**	205.1±34.6	202.9±34.3	204.9±33.9	205.6±34.6	207.2±35.6	<0.001
**Triglyceride (mg/dL)**	132 (95–185)	104 (77–144)	127 (93–173)	140 (103–192)	161 (118–224)	<0.001
**LDL (mg/dL)**	134.2±31.3	131.9±31.6	133.9±30.7	134.8±31.0	135.9±31.9	<0.001
**HDL (mg/dL)**	49.5±11.3	52.7±12.1	50.1±11.4	48.7±10.7	46.5±10.1	<0.001
**Antidyslipidemic medication, n (%)**	740 (2.27)	146 (1.8)	182 (2.23)	191 (2.35)	221 (2.7)	0.002
**Dyslipidemia, n (%)**	4,717 (14.5)	975 (12.0)	1,056 (12.9)	1,273 (15.6)	1,413 (17.3)	<0.001
**hs-CRP (mg/dL)**	0.14±0.32	0.12±0.29	0.13±0.29	0.14±0.33	0.18±0.37	<0.001
**Current alcohol use^*b*^, n (%)**	15,459 (47.4)	3,790 (46.7)	3,866 (47.4)	3,861 (47.4)	3,942 (48.1)	0.367
** Male**	12,786 (47.2)	3,186 (46.1)	3,284 (47.2)	3,253 (47.3)	3,063 (48.2)	
** Female**	2,673 (48.5)	604 (50.3)	582 (48.0)	608 (48.1)	879 (47.8)	
**Smoking status, n (%)**						<0.001
** Never**	11,881 (36.4)	2,666 (32.9)	2,861 (35.0)	2,987 (36.7)	3,367 (41.1)	
** Ex**	10,024 (30.7)	2,680 (33.1)	2,514 (30.8)	2,504 (30.8)	2,326 (28.4)	
** Current**	9,660 (29.6)	2,449 (30.2)	2,535 (31.1)	2,399 (29.5)	2,277 (27.8)	
**Regular exercise, n (%)**						<0.001
** ≥3 times/week**	3,581 (11.0)	1,097 (13.5)	908 (11.1)	827 (10.2)	749 (9.1)	
** <3 times/week**	28,723 (88.1)	6,921 (85.4)	7,185 (88.0)	7,238 (88.9)	7,379 (90.1)	
**APRI**	0.25±0.08	0.25±0.08	0.25±0.08	0.25±0.08	0.26±0.09	<0.001

Abbreviations: BMI, body mass index; WC, waist circumference; FBG, fasting blood glucose; SBP, systolic blood pressure; AST, aspartate aminotransferase; ALT, alanine aminotransferase; LDL, low-density lipoprotein; HDL, high-density lipoprotein; hs-CRP, high-sensitivity C-reactive protein; APRI, aspartate aminotransferase-to-platelet ratio index.

^*a*^Obesity was defined as a BMI ≥25 kg/m^2^.

^*b*^Participants with daily alcohol consumption above the median value (11 g/day for men and 2 g/day for women)

### Relationship between the baseline HOMA-IR and the development of advanced liver fibrosis

**Fig 2:**
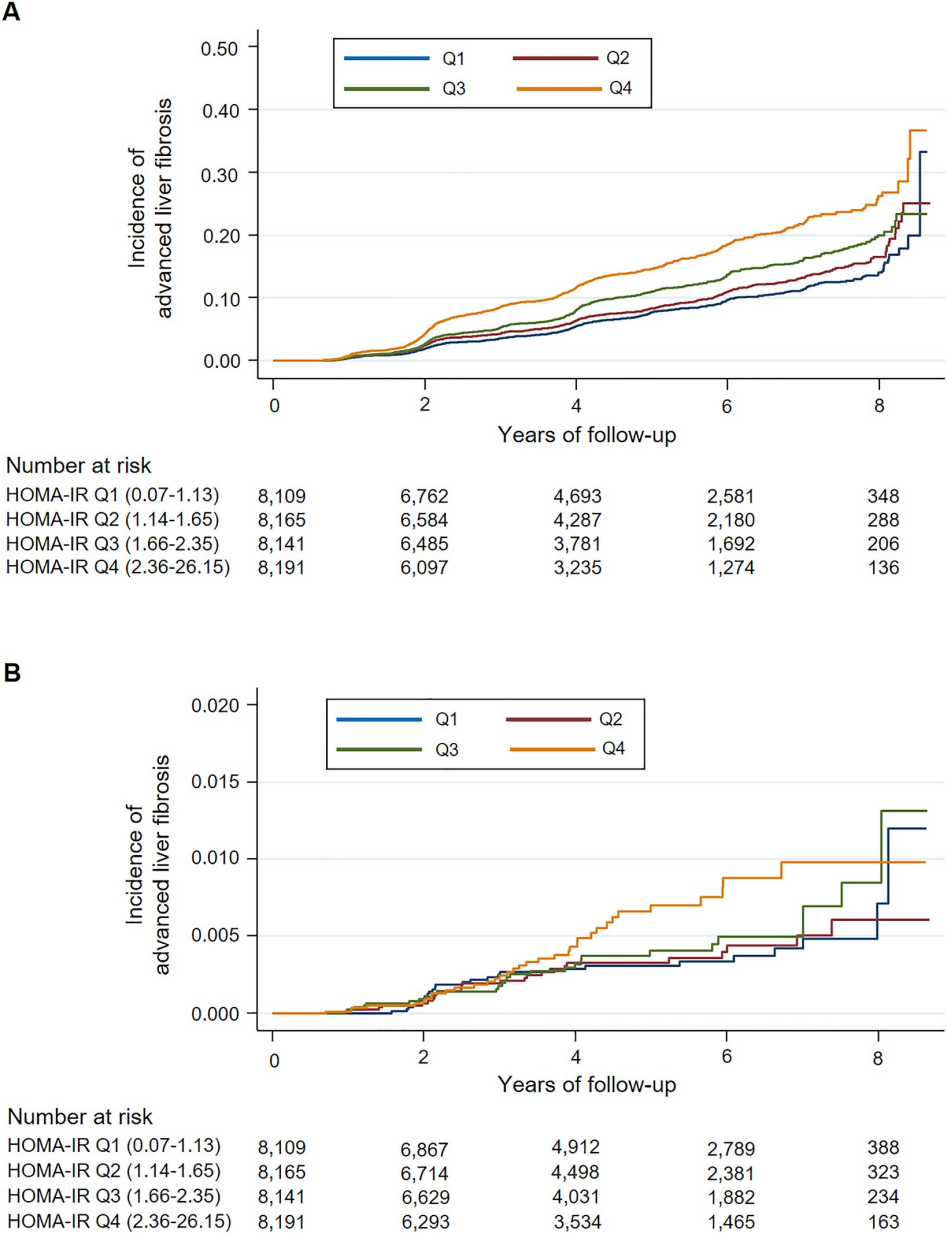
Cumulative incidence of advanced liver fibrosis by HOMA-IR quartile at baseline. (A) Above the intermediate fibrosis probability (APRI ≥0.5), (B) With high fibrosis probability (APRI >1.5). Abbreviations: HOMA-IR, homeostatic model assessment of insulin resistance; APRI, aspartate aminotransferase-to-platelet ratio index.

In Table 2, advanced liver fibrosis (APRI ≥0.5) developed in a total of 2,897 participants during 136,108 person-years (median follow-up, 3.97 years). In all models, as the baseline HOMA-IR increased, the HR tended to increase. The HR for advanced liver fibrosis in group Q4 relative to group Q1 was 2.18 (95% CI 1.96–2.42) in Model 1. However, the HR decreased to 1.95 (95% CI 1.74–2.19; Model 4) after adjusting for confounding variables mentioned above. The cumulative incidence of advanced liver fibrosis with an intermediate or high probability linearly increased in all quartile groups from the 2nd year of follow-up. Subsequently, the positive association between baseline HOMA-IR and the incidence of advanced liver fibrosis was maintained throughout the entire follow-up period (Fig 2A).

**Table 2:** Hazard ratios for the development of advanced liver fibrosis by HOMA-IR quartile at baseline.

HOMA-IR quartile	No.^*a*^	Cases	IR (per 10^4^ PY)	Model 1 HR (95% CI)	Model 2 HR (95% CI)	Model 3 HR (95% CI)	Model 4 HR (95% CI)
**Above the intermediate fibrosis probability (APRI ≥0.5)**							
** Total**	32,606	2,897	212.8 (205.2–220.7)				
** Q1 (0.07–1.13)**	8,109	596	159.4 (147.1–172.7)	1 (reference)	1 (reference)	1 (reference)	1 (reference)
** Q2 (1.14–1.65)**	8,165	651	183.4 (169.9–198.1)	1.18 (1.06–1.32)	1.15 (1.03–1.28)	1.10 (0.98–1.23)	1.14 (1.02–1.28)
** Q3 (1.66–2.35)**	8,141	724	219.5 (204.1–236.1)	1.48 (1.32–1.65)	1.41 (1.27–1.58)	1.30 (1.16–1.46)	1.40 (1.25–1.57)
** Q4 (2.36–26.15)**	8,191	926	305.8 (286.8–326.2)	2.18 (1.96–2.42)	2.00 (1.80–2.23)	1.71 (1.52–1.92)	1.95 (1.74–2.19)
** *p*-value**				<0.001	<0.001	<0.001	<0.001
**With high fibrosis probability (APRI >1.5)**							
** Total**	32,606	114	8.0 (6.7–9.7)				
** Q1 (0.07–1.13)**	8,109	26	6.7 (4.5–9.9)	1 (reference)	1 (reference)	1 (reference)	1 (reference)
** Q2 (1.14–1.65)**	8,165	25	6.8 (4.6–10.1)	1.02 (0.59–1.77)	1.03 (0.59–1.78)	0.95 (0.54–1.69)	0.99 (0.56–1.76)
** Q3 (1.66–2.35)**	8,141	28	8.1 (5.6–11.8)	1.25 (0.73–2.14)	1.26 (0.74–2.17)	1.25 (0.72–2.18)	1.34 (0.77–2.34)
** Q4 (2.36–26.15)**	8,191	35	11.0 (7.9–15.3)	1.73 (1.03–2.92)	1.76 (1.03–3.00)	1.70 (0.96–3.01)	1.95 (1.10–3.46)
** *p*-value**				0.027	0.028	0.044	0.014

Model 1 adjusted for age, sex, and year of examination. Model 2 was additionally adjusted for SBP, antihypertensive medications, regular exercise, current alcohol consumption, and smoking status. Model 3 was additionally adjusted for BMI, waist circumference, HbA1c, hs-CRP, LDL cholesterol, HDL cholesterol, triglyceride, and lipid-lowering medications. Model 4, additionally adjusted for time-varying development of diabetes and HOMA-IR change during the follow-up period.

Abbreviations: HOMA-IR, homeostasis model assessment of insulin resistance; IR, incidence rate; PY, person-years; HR, hazard ratio; APRI, aspartate aminotransferase-to-platelet ratio index; SBP, systolic blood pressure; BMI, body mass index; HbA1c, hemoglobin A1c; hs-CRP, high-sensitivity C-reactive protein; LDL, low-density lipoprotein; HDL, high-density lipoprotein.

^*a*^ Number of participants.

However, only 114 participants developed advanced liver fibrosis with high probability (APRI >1.5) during 141,064 person-years (median follow-up, 4.06 years) (Table 2). When compared with group Q1 as a reference, the HR for advanced liver fibrosis with high fibrosis probability in group Q4 was 1.73 (95% CI 1.03–2.92) in Model 1. This value was nearly maintained in Models 2 and 3, but slightly increased to 1.95 (95% CI 1.10–3.46) in Model 4. As shown in Fig 2B, the overall tendency in the cumulative incidence of advanced liver fibrosis with high probability was similar to that of Fig 2A. However, meaningful differences in the incidence of advanced liver fibrosis between quartile groups began to appear from the 4th year of follow-up relatively.

The optimal cut-off value of baseline HOMA-IR as a surrogate marker for predicting the development of advanced liver fibrosis was 1.68 (sensitivity 56.2%, specificity 51.5%, positive predictive value 10.2%, and negative predictive value 92.3%), which was obtained using Youden’s J index and area under the receiver operating characteristic (ROC) curve (AUC 0.553, 95% CI; 0.541–0.564) (S1 Fig) [33].

### Subgroup analysis

**Fig 3:**
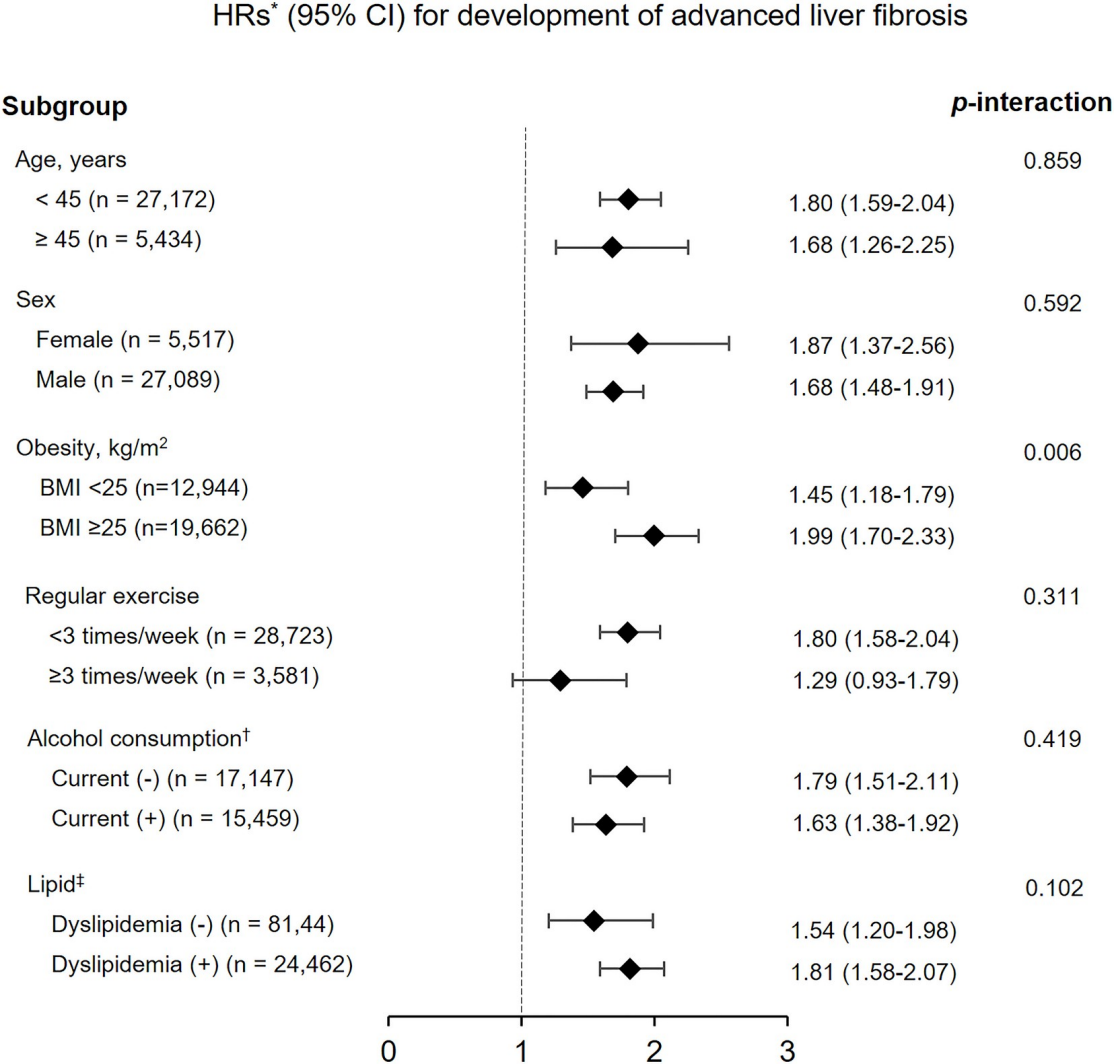
Subgroup analyses of hazard ratios for the development of advanced liver fibrosis (APRI value of ≥0.5) in participants belonging to the highest baseline HOMA-IR quartile (Q4) compared with those belonging to the lowest baseline HOMA-IR quartile (Q1). *Adjusted for age, sex, year of examination, SBP, antihypertensive medications, regular exercise, current alcohol consumption, smoking status, BMI, waist circumference, HbA1c, hs-CRP, LDL cholesterol, HDL cholesterol, triglyceride, and use of antidyslipidemic drugs. ^†^ Current alcohol consumption defined as daily alcohol consumption above the median value (12 g/day for men, 2 g/day for women). ^‡^ Dyslipidemia was defined as total cholesterol ≥200 mg/dL, triglyceride levels ≥150 mg/dL, LDL cholesterol levels ≥130 mg/dL, HDL cholesterol levels <40 mg/dL in men and <50 mg/dL in women, or use of antidyslipidemic drugs. Abbreviations: HOMA-IR, homeostasis model assessment of insulin resistance; SBP, systolic blood pressure; BMI, body mass index; HbA1c, hemoglobin A1c; hs-CRP, high-sensitivity C-reactive protein; LDL, low-density lipoprotein; HDL, high-density lipoprotein; HR, hazard ratio; CI, confidence interval.

In all subgroup analyses, the HRs for development of advanced liver fibrosis with an APRI value of ≥0.5 also tended to increase as the baseline HOMA-IR quartile increased. All p-values for interaction were >0.05, except obesity (p-interaction = 0.006) (Fig 3). In the analysis of advanced liver fibrosis with high probability, all p-values for interaction were >0.05, and group Q4 was associated with an increased incidence of advanced liver fibrosis except in participants who exercised regularly (≥3 times/week) (S2 Table).

### Comparison of baseline HOMA-IR and baseline BMI values with respect to the prediction of fibrosis probability progression

S3 Table demonstrates the comparison between standardized quartiles of baseline HOMA-IR and standardized quartiles of baseline BMI with respect to the risk of advanced liver fibrosis in patients with NAFLD without diabetes. There was no significant difference in quantitative influence; the increase in HR per 1 SD of baseline HOMA-IR values was 1.13 (95% CI 1.10–1.15), and the increase in HR per 1 SD of baseline BMI values was 1.16 (95% CI 1.08–1.25). In the predictive validity analysis for the development of advanced liver fibrosis with an APRI value of ≥0.5, the baseline HOMA-IR model showed a smaller AIC value than the baseline BMI model (54,387 vs. 54,449.29). For the analysis of advanced liver fibrosis with high fibrosis probability (APRI >1.5), the baseline BMI model was not statistically significant.

## Discussion

In this longitudinal cohort study of 32,606 young and middle-aged adult participants, we found that higher quartiles of baseline HOMA-IR, i.e., higher insulin resistance, were associated with higher risk of fibrosis progression as evaluated by APRI value in participants with NAFLD without diabetes. In addition, this positive association remained constant throughout the follow-up period. This suggests that insulin resistance at the time of NAFLD diagnosis may be a key factor in future fibrosis progression. This tendency was maintained in all models adjusted for risk factors associated with the development of NAFLD or fibrosis progression and remained even when we eliminated the effect of diabetes that developed after baseline. The results were also consistent in predefined subgroup analysis.

NAFLD is a broad-spectrum disease that can simultaneously or separately show steatosis, NASH, fibrosis, and cirrhosis [34]. In addition to IR, activation of inflammation due to an adverse lifestyle such as a high-calorie diet, genetic factors associated with family history, oxidative stress, and lipotoxicity are the underlying mechanisms of the pathogenesis of NAFLD and progressive fibrosis. IR interferes with the anti-lipolytic effect of insulin and increases free fatty acids (FFA). These excess FFA enter the liver and accumulate. Moreover, increased insulin levels promote triglyceride (TG) synthesis in hepatocytes. In this process, very low density lipoprotein production is activated and increased TG is also a source of FFA. This steatosis is associated with oxidative stress, lipid peroxidation, and increased secretion of inflammatory markers. FFA also stimulate the production of cytokines and chemokines by activating Toll-like receptors (TLRs) and inflammasomes. In the fibrosis of NAFLD, Kupffer cells, recruited macrophages and stellate cells are key regulators activated by cytokines, chemokines, and oxidative stress [35, 36]. In these processes, hepatic mitochondrial dysfunction and triacylglycerol accumulation are included [37], and IR, lipotoxicity, and inflammation influence each other to form a vicious cycle and at the same time to further promote fibrosis progression in patients with NAFLD [38].

A prospective study of patients with NAFLD demonstrated that baseline HOMA-IR, which reflects an estimate of baseline IR, is an independent risk factor for advanced fibrosis [19]. In 2 studies on the progression of fibrosis in patients with NAFLD based on serial biopsies at intervals of ≥1 year, a tendency to have higher baseline HOMA-IR was confirmed in patients with fibrosis progression (5.8±5.1 SD vs. 3.9±2.5 SD, *p* = 0.09) [20]. Increased IR was also shown to play a key role in the progression of simple steatosis to steatohepatitis with fibrosis [39]. In a prospective cohort study analyzing the association between weight change and the risk of fibrosis progression as evaluated by APRI values in 40,700 Korean adults with NAFLD, the baseline HOMA-IR value was a risk factor for fibrosis progression [24]. Interestingly, in that study, the influence of weight change on fibrosis progression depended on baseline HOMA-IR value, i.e., it was greater in patients with HOMA-IR <2.5. Recently, in a retrospective study using histological verification in patients with NAFLD, the HOMA-IR value at the time of evaluation was closely associated with the degree of liver fibrosis at the same time in patients with NAFLD regardless of the presence of diabetes [21–23].

Obesity, one of the most important phenotypes of patients with metabolic syndrome, is also closely associated with the aggravation of IR and development of NAFLD; obesity and NAFLD share many common pathophysiological mechanisms [35, 40]. Increases in waist circumference and visceral-subcutaneous abdominal fat ratio have been reported to be associated with the development of NAFLD and advanced liver fibrosis [41, 42]. A prospective Korean cohort study showed that weight gain and baseline obesity were associated with APRI progression in patients with NAFLD [24]. These findings are consistent with the results of our study. In our study, the HRs for the development of advanced liver fibrosis above the intermediate fibrosis risk according to baseline HOMA-IR quartile were affected by baseline obesity status (*p* for interaction = 0.006). In other words, the presence or absence of obesity influenced the association between baseline HOMA-IR value and the risk of advanced liver fibrosis, and the positive association was more pronounced in participants with obesity (BMI ≥25 kg/m^2^). Therefore, we performed a comparative analysis of quantitative power and prediction quality for the development of advanced liver fibrosis, defined as an APRI value of ≥0.5, between standardized baseline HOMA-IR values and standardized baseline BMI values. Interestingly, based on the AIC calculation, the baseline HOMA-IR model was superior to the baseline BMI model in predicting the advanced liver fibrosis as evaluated by the APRI. However, the quantitative increase in HR per 1 SD was similar in both models.

Despite these statistically significant results, there are some limitations to the interpretation. First, in the diagnosis of NAFLD and fibrosis stage, histological evaluation was not performed, which is a practical limitation of large-scale cohort studies due to the low clinical utility of biopsies in the general population [17]. Despite some of its disadvantages, USG has a relatively high accuracy in diagnosing NAFLD [43]. In addition, the scoring systems, such as NFS and FIB-4, using metabolic variables for predicting the fibrosis progression of NAFLD provide convenience and efficiency in large-scale cohort studies and provide clinically acceptable positive and negative predictive values [7, 44]. However, NFS includes age and BMI as components. And FIB-4 also includes age as a component. In longitudinal studies like ours, age itself can influence outcomes [24]. To exclude the effect of BMI, BMI along with age was included as an adjustment factor. However, if NFS is applied, age and BMI cannot be included as adjustment factors. In addition, NFS cannot be applied in analysis to compare baseline HOMA-IR and baseline BMI. We performed the same analysis based on NFS and FIB-4, and found no statistical significance (No data was provided). APRI could be used as a useful diagnostic tool in the evaluation of fibrosis in patients with NAFLD in a longitudinal study [24, 31, 45]. Nevertheless, in our longitudinal study, baseline HOMA-IR is not a tool to directly diagnose liver fibrosis, and we chronologically analyzed the association between baseline HOMA-IR and incidence of future advanced liver fibrosis indirectly assessed by APRI progression. So, the baseline HOMA-IR cut-off value obtained using AUC requires caution in applying and interpreting the diagnosis of advanced liver fibrosis due to the limitation of applying the APRI ≥0.5 criterion. Second, among the data used in the analysis of our study, the lifestyle, medications, and past medical history, other than laboratory test and measurement data, completely depend on information provided by the participants without any other verification procedure. Third, the effects of drugs and dietary supplements other than antidiabetic, anti-dyslipidemia, and antihypertensive agents were not excluded. Finally, most study participants were relatively young white-collar Korean workers, and the majority of them were men (83%). Sex, race, and age are factors that influence the development and progression of NAFLD [46, 47]. Although age and sex were adjusted for in the analysis of HRs and cumulative incidence of advanced liver fibrosis, the results of our study have limited generalizability. Nonetheless, our study has clinical implications as a highly sophisticated longitudinal study that assessed the progression of fibrosis probability using a non-invasive scoring system according to the baseline HOMA-IR value in participants with NAFLD without diabetes based on a large cohort data.

In conclusion, this study demonstrated that baseline IR assessed using HOMA-IR in patients with NAFLD without diabetes was associated with the future development of advanced liver fibrosis, i.e., the higher the baseline HOMA-IR, the higher the incidence of fibrosis probability progression.

## Supporting information

S1 Table Baseline characteristics of participants by development of advanced liver fibrosis (APRI ≥0.5). (DOCX)

S2 Table Subgroup analyses of hazard ratios for development of advanced liver fibrosis with high probability (APRI >1.5) according to HOMA-IR quartile at baseline. (DOCX)

S3 Table Comparison between standardized quartiles of baseline HOMA-IR and standardized quartiles of baseline BMI for the risk of advanced liver fibrosis in patients with NAFLD without diabetes. (DOCX)

S1 Fig AUC for the optimal cut-off value of baseline HOMA-IR for predicting future development of advanced liver fibrosis (APRI ≥0.5) in patients with NAFLD without diabetes. (TIF)

## Abbreviations

IR: insulin resistance
NAFLD: non-alcoholic fatty liver disease
NASH: nonalcoholic steatohepatitis
HOMA–IR: homeostasis model assessment of insulin resistance
APRI: AST to platelet ratio index
BMI: body mass index
SBP: systolic blood pressure
DBP: diastolic blood pressure
AST: aspartate transaminase
hs-CRP: high-sensitivity C-reactive protein
HDL: high-density lipoprotein
LDL: low-density lipoprotein
HR: hazard ratio
SD: standard deviation
AUC: area under the receiver operating characteristic curve
