# Supplementary material for: Baseline homeostasis model assessment of insulin resistance associated with fibrosis progression in patients with nonalcoholic fatty liver disease without diabetes: A cohort study
Source: PLoS One. 2021 Aug 25;16(8):e0255535. doi: 10.1371/journal.pone.0255535 (PMC8386882; doi:10.1371/journal.pone.0255535)
Supplement: S1 Table — (DOCX) [file pone.0255535.s001.docx]

**S1 Table. Baseline characteristics of participants by development of advanced liver fibrosis (APRI ≥0.5).**

| **Characteristics** | **Overall** | **Advanced liver fibrosis (APRI ≥0.5)** | | ***p*-value** |
| --- | --- | --- | --- | --- |
|  |  | **No** | **Yes** |  |
| Number of participants | 32,606 | 29,709 | 2,897 |  |
| Male, n (%) | 27,089 (83.08) | 24,610 (82.84) | 2,479 (85.57) | <0.001 |
| Age, years | 37.95±7.73 | 37.96±7.72 | 37.89±7.8 | <0.001 |
| BMI, kg/m^2^ | 26.01±3.04 | 25.95±3.02 | 26.58±3.15 | <0.001 |
| Obesity^a^, n (%) | 19,662 (60.3) | 17,707 (59.6) | 1,955 (67.48) | <0.001 |
| Waist circumference, cm | 89.14±7.79 | 89.01±7.76 | 90.51±7.96 | <0.001 |
| Fasting blood glucose, mg/dL | 95.49±9 | 95.43±8.97 | 96.12±9.3 | <0.001 |
| Hemoglobin A1c, % | 5.61±0.29 | 5.61±0.29 | 5.63±0.3 | <0.001 |
| SBP, mmHg | 115.82±12.09 | 115.66±12.09 | 117.52±12.05 | <0.001 |
| Antihypertensive medications, n (%) | 1,827 (5.6) | 1,636 (5.51) | 191 (6.59) | 0.015 |
| History of hypertension, n (%) | 3,637 (11.15) | 3,264 (10.99) | 373 (12.88) | 0.002 |
| AST, IU/L | 23 (19–28) | 22 (19–27) | 28 (22–34) | <0.001 |
| ALT, IU/L | 28 (20–40) | 27 (20–39) | 37 (25–53) | <0.001 |
| Platelets, ×10^3^/mm^3^ | 255.65±49.82 | 257.51±49.73 | 236.56±46.63 | <0.001 |
| Albumin, g/dL | 4.7 (4.5–4.9) | 4.7 (4.5–4.9) | 4.7 (4.5–4.9) | 0.008 |
| Total cholesterol, mg/dL | 205.14±34.64 | 204.91±34.56 | 207.51±35.31 | <0.001 |
| Triglyceride, mg/dL | 132 (95–185) | 131 (94–182) | 149 (104–212) | <0.001 |
| LDL, mg/dL | 134.15±31.32 | 134.15±31.25 | 134.14±32 | <0.001 |
| HDL, mg/dL | 49.5±11.33 | 49.63±11.3 | 48.23±11.47 | <0.001 |
| Lipid lowering medication, n (%) | 740 (2.27) | 642 (2.16) | 98 (3.38) | <0.001 |
| History of dyslipidemia, n (%) | 4,717 (14.47) | 4,241 (14.28) | 476 (16.43) | 0.002 |
| hs-CRP, mg/dL | 0.14±0.32 | 0.14±0.33 | 0.14±0.3 | <0.001 |
| Current alcohol use^b^, n (%) | 15,459 (47.41) | 14,003 (47.13) | 1,456 (50.26) | 0.001 |
| Male | 12,786 (47.2) | 11,520 (46.81) | 1,266 (51.07) | <0.001 |
| Female | 2,673 (48.45) | 2,483 (48.7) | 190 (45.45) | 0.202 |
| Smoking status, n (%) |  |  |  | <0.001 |
| Never | 11,881 (36.44) | 10,944 (36.84) | 937 (32.34) |  |
| Ex | 10,024 (30.74) | 9,113 (30.67) | 911 (31.45) |  |
| Current | 9,660 (29.63) | 8,719 (29.35) | 941 (32.48) |  |
| Regular exercise, n (%) |  |  |  | 0.014 |
| ≥3 times/week | 2,8723 (88.09) | 26,217 (88.25) | 2,506 (86.5) |  |
| <3 times/week | 3,581 (10.98) | 3,216 (10.83) | 365 (12.6) |  |
| APRI | 0.25±0.08 | 0.25±0.08 | 0.32±0.09 | <0.001 |
| HOMA-IR | 1.66 (1.14–2.36) | 1.64 (1.13–2.33) | 1.81 (1.22–2.64) | <0.001 |

Abbreviations: BMI, body mass index; SBP, systolic blood pressure; AST, aspartate aminotransferase; ALT, alanine aminotransferase; LDL, low-density lipoprotein; HDL, high-density lipoprotein; hs-CRP, high-sensitivity C-reactive protein; APRI, aspartate aminotransferase-to-platelet ratio index.

^a^ Obesity was defined as a BMI ≥25 kg/m^2^.

^b^ Participants with daily alcohol consumption above the median value (11 g/day for men and 2 g/day for women).
