# Supplementary material for: Baseline homeostasis model assessment of insulin resistance associated with fibrosis progression in patients with nonalcoholic fatty liver disease without diabetes: A cohort study
Source: PLoS One. 2021 Aug 25;16(8):e0255535. doi: 10.1371/journal.pone.0255535 (PMC8386882; doi:10.1371/journal.pone.0255535)
Supplement: S2 Table — (DOCX) [file pone.0255535.s002.docx]

**S2 Table. Subgroup analyses of hazard ratios for development of advanced liver fibrosis with high probability (APRI >1.5) according to HOMA-IR quartile at baseline.**

| **Subgroups** | **HOMA-IR quartile** | **No^a^** | **Cases** | **IR (per 10^4^ PY)** | **Multivariate adjusted^b^ HR (95% CI)** |
| --- | --- | --- | --- | --- | --- |
| Age |  |  |  |  |  |
| <45 years | Q1 | 6,366 | 24 | 7.68 (5.15–11.46) | 1 (reference) |
|  | Q2 | 6,703 | 21 | 6.84 (4.46–10.49) | 0.896 (0.49–1.639) |
|  | Q3 | 6,912 | 24 | 8.12 (5.44–12.12) | 1.156 (0.644–2.077) |
|  | Q4 | 7,191 | 32 | 11.35 (8.03–16.05) | 1.659 (0.918–2.999) |
| ≥45 years | Q1 | 1,743 | 2 | 2.75 (0.69–10.98) | reference |
|  | Q2 | 1,462 | 4 | 6.77 (2.54–18.04) | 1.868 (0.305–11.431) |
|  | Q3 | 1,229 | 4 | 8.56 (3.21–22.81) | 2.778 (0.476–16.21) |
|  | Q4 | 1,000 | 3 | 8.55 (2.76–26.5) | 3.448 (0.479–24.8) |
| *p*-interaction |  |  |  |  | 0.737 |
| Sex |  |  |  |  |  |
| Female | Q1 | 1,202 | 1 | 1.96 (0.28–13.9) | 1 (reference) |
|  | Q2 | 1,213 | 3 | 5.93 (1.91–18.38) | 1.9 (0.17–21) |
|  | Q3 | 1,264 | 2 | 3.88 (0.97–15.52) | 1.67 (0.15–18.79) |
|  | Q4 | 1,838 | 11 | 15.95 (8.83–28.8) | 5.76 (0.67–49.96) |
| Male | Q1 | 6907 | 25 | 7.48 (5.05–11.07) | reference |
|  | Q2 | 6,952 | 22 | 6.97 (4.59–10.59) | 0.94 (0.52–1.71) |
|  | Q3 | 6,877 | 26 | 8.94 (6.09–13.14) | 1.31 (0.74–2.32) |
|  | Q4 | 6,353 | 24 | 9.67 (6.48 14.43) | 1.48 (0.8–2.77) |
| *p*-interaction |  |  |  |  | 0.165 |
| Obesity (kg/m^2^) |  |  |  |  |  |
| BMI <25 | Q1 | 4,905 | 18 | 7.79 (4.91–12.36) | 1 (reference) |
|  | Q2 | 3,621 | 16 | 9.87 (6.05–16.12) | 1.32 (0.65–2.66) |
|  | Q3 | 2,798 | 10 | 8.58 (4.62–15.95) | 1.23 (0.55–2.73) |
|  | Q4 | 1,620 | 9 | 13.99 (7.28–26.89) | 2.10 (0.90–4.90) |
| BMI ≥25 | Q1 | 3,204 | 8 | 5.19 (2.6–10.38) | 1 (reference) |
|  | Q2 | 4,544 | 9 | 4.41 (2.29–8.47) | 0.75 (0.28–2.00) |
|  | Q3 | 5,343 | 18 | 7.98 (5.03–12.66) | 1.51 (0.65–3.52) |
|  | Q4 | 6,571 | 26 | 10.29 (7–15.11) | 1.76 (0.75–4.11) |
| *p*-interaction |  |  |  |  | 0.605 |
| Exercise status |  |  |  |  |  |
| <3 times/week | Q1 | 6,921 | 19 | 5.75 (3.67–9.01) | 1 (reference)e |
|  | Q2 | 7,185 | 22 | 6.81 (4.48–10.34) | 1.137 (0.599–2.158) |
|  | Q3 | 7,238 | 21 | 6.89 (4.49–10.56) | 1.281 (0.673–2.439) |
|  | Q4 | 7,379 | 33 | 11.54 (8.2–16.23) | 2.168 (1.15–4.085) |
| ≥3 times/week | Q1 | 1,097 | 7 | 13.78 (6.57–28.9) | 1 (reference) |
|  | Q2 | 908 | 3 | 7.5 (2.42–23.24) | 0.535 (0.135–2.123) |
|  | Q3 | 827 | 7 | 20.71 (9.87–43.44) | 1.487 (0.474–4.666) |
|  | Q4 | 749 | 2 | 7.08 (1.77–28.31) | 0.589 (0.107–3.254) |
| *p*-interaction |  |  |  |  | 0.224 |
| Alcohol consumption^c^ |  |  |  |  |  |
| Current (-) | Q1 | 4,319 | 17 | 8.17 (5.08–13.14) | 1 (reference) |
|  | Q2 | 4,299 | 14 | 7.16 (4.24–12.09) | 0.813 (0.382–1.728) |
|  | Q3 | 4,280 | 11 | 6.08 (3.37–10.97) | 0.814 (0.369–1.793) |
|  | Q4 | 4,249 | 13 | 7.8 (4.53–13.43) | 1.077 (0.476–2.436) |
| Current (+) | Q1 | 3,790 | 9 | 5.08 (2.64–9.76) | 1 (reference) |
|  | Q2 | 3,866 | 11 | 6.45 (3.57–11.64) | 1.246 (0.513–3.024) |
|  | Q3 | 3,861 | 17 | 10.55 (6.56–16.97) | 2.042 (0.893–4.668) |
|  | Q4 | 3,942 | 22 | 14.64 (9.64–22.23) | 2.823 (1.208–6.596) |
| *p*-interaction |  |  |  |  | 0.215 |
| Lipid^d^ |  |  |  |  |  |
| Dyslipidemia (-) | Q1 | 2,862 | 5 | 3.71 (1.54–8.9) | 1 (reference) |
|  | Q2 | 2,141 | 4 | 4.23 (1.59–11.27) | 0.575 (0.11–3) |
|  | Q3 | 1,813 | 4 | 5.23 (1.96–13.94) | 1.463 (0.377–5.674) |
|  | Q4 | 1,328 | 5 | 9.51 (3.96–22.85) | 2.796 (0.695–11.254) |
| Dyslipidemia (+) | Q1 | 5,247 | 21 | 8.39 (5.47–12.87) | 1 (reference) |
|  | Q2 | 6,024 | 21 | 7.73 (5.04–11.86) | 0.97 (0.524–1.797) |
|  | Q3 | 6,328 | 24 | 9.03 (6.05–13.48) | 1.147 (0.624–2.107) |
|  | Q4 | 6,863 | 30 | 11.34 (7.93–16.22) | 1.478 (0.793–2.753) |
| *p*-interaction |  |  |  |  | 0.622 |

Abbreviations: APRI, aspartate aminotransferase-to-platelet ratio index; HOMA-IR, homeostasis model assessment of insulin resistance; IR, incidence rate; PY, person-years; HR, hazard ratio; SBP, systolic blood pressure; BMI, body mass index; HbA1c, hemoglobin A1c; hs-CRP, high-sensitivity C-reactive protein; LDL, low-density lipoprotein; HDL, high-density lipoprotein.

^a^ Number of participants.

^b^ Adjusted for age, sex, year of examination, SBP, antihypertensive medications, regular exercise, current alcohol consumption, smoking status, BMI, waist circumference, HbA1c, hs-CRP, LDL cholesterol, triglyceride, use of antidyslipidemic drugs.

^c^ Current alcohol consumption was defined as daily alcohol consumption above the median value (12 g/day for men and 2 g/day for women).

^d^ Dyslipidemia was defined as total cholesterol ≥200 mg/dL, triglyceride levels ≥150 mg/dL, LDL cholesterol levels ≥130 mg/dL, HDL cholesterol levels <40 mg/dL in men and <50 mg/dL in women, or use of antidyslipidemic drugs.
