# Supplementary material for: Baseline homeostasis model assessment of insulin resistance associated with fibrosis progression in patients with nonalcoholic fatty liver disease without diabetes: A cohort study
Source: PLoS One. 2021 Aug 25;16(8):e0255535. doi: 10.1371/journal.pone.0255535 (PMC8386882; doi:10.1371/journal.pone.0255535)
Supplement: S3 Table — (DOCX) [file pone.0255535.s003.docx]

**S3 Table. Comparison between standardized quartiles of baseline HOMA-IR and standardized quartiles of baseline BMI for the risk of advanced liver fibrosis in patients with NAFLD without diabetes.**

| **Standardized quartile** | **Multivariable-adjusted HR^a^ (95% CI)** | |
| --- | --- | --- |
|  | **Baseline HOMA-IR^b^** | **Baseline BMI^c^** |
| Above the intermediate fibrosis probability (APRI ≥0.5) |  |  |
| Q1 | 1 (reference) | 1 (reference) |
| Q2 | 1.19 (1.01–1.40) | 0.99 (0.86–1.13) |
| Q3 | 1.50 (1.26–1.77) | 1.09 (0.94–1.28) |
| Q4 | 2.15 (1.79–2.59) | 1.29 (1.06–1.58) |
| *p* for trend | <0.001 | 0.003 |
| Per 1 SD increase | 1.13 (1.10–1.15) | 1.16 (1.08–1.25) |
| Akaike information criterion | 54,387.92 | 54,449.29 |
| With high fibrosis probability (APRI >1.5) |  |  |
| Q1 | 1 (reference) | 1 (reference) |
| Q2 | 1.39 (0.59–3.25) | 1.01 (0.54–1.90) |
| Q3 | 1.93 (0.79–4.70) | 0.68 (0.32–1.47) |
| Q4 | 2.69 (1.01–7.17) | 0.86 (0.31–2.33) |
| p for trend | 0.01 | 0.404 |
| Per 1 SD increase | 1.11 (0.98–1.25) | 0.80 (0.54–1.17) |
| Akaike information criterion | 2,174.67 | 2,175.17 |

Abbreviations: HOMA-IR, homeostasis model assessment of insulin resistance; BMI, body mass index; NAFLD, nonalcoholic fatty liver disease; HR, hazard ratio; CI, confidence interval; SBP, systolic blood pressure; hs-CRP, high-sensitivity C-reactive protein; SD, standard deviation.

^a^ Adjusted for age, sex, year of examination, SBP, antihypertensive medications, regular exercise, current alcohol consumption, smoking status, waist circumference, HbA1c, hs-CRP, LDL cholesterol, triglyceride, use of antidyslipidemic drugs.

^b^ Standardized quartile range of baseline HOMA–IR: Q1, 0.07–0.74 (n=2,501); Q2, 0.74–1.91 (n=17,157); Q3, 1.91–3.09 (n=8,984); Q4, 3.09–26.15 (n=3,964).

^c^ Standardized quartile range of baseline BMI: Q1, 15.47–22.97 (n=4,456); Q2, 22.97–26.01 (n=13,335); Q3, 26.01–29.04 (n=10,147); Q4, 29.04–47.41 (n=4,668).
