# Supplementary figures and images for: Baseline homeostasis model assessment of insulin resistance associated with fibrosis progression in patients with nonalcoholic fatty liver disease without diabetes: A cohort study
Source: PLoS One. 2021 Aug 25;16(8):e0255535. doi: 10.1371/journal.pone.0255535 (PMC8386882; doi:10.1371/journal.pone.0255535)

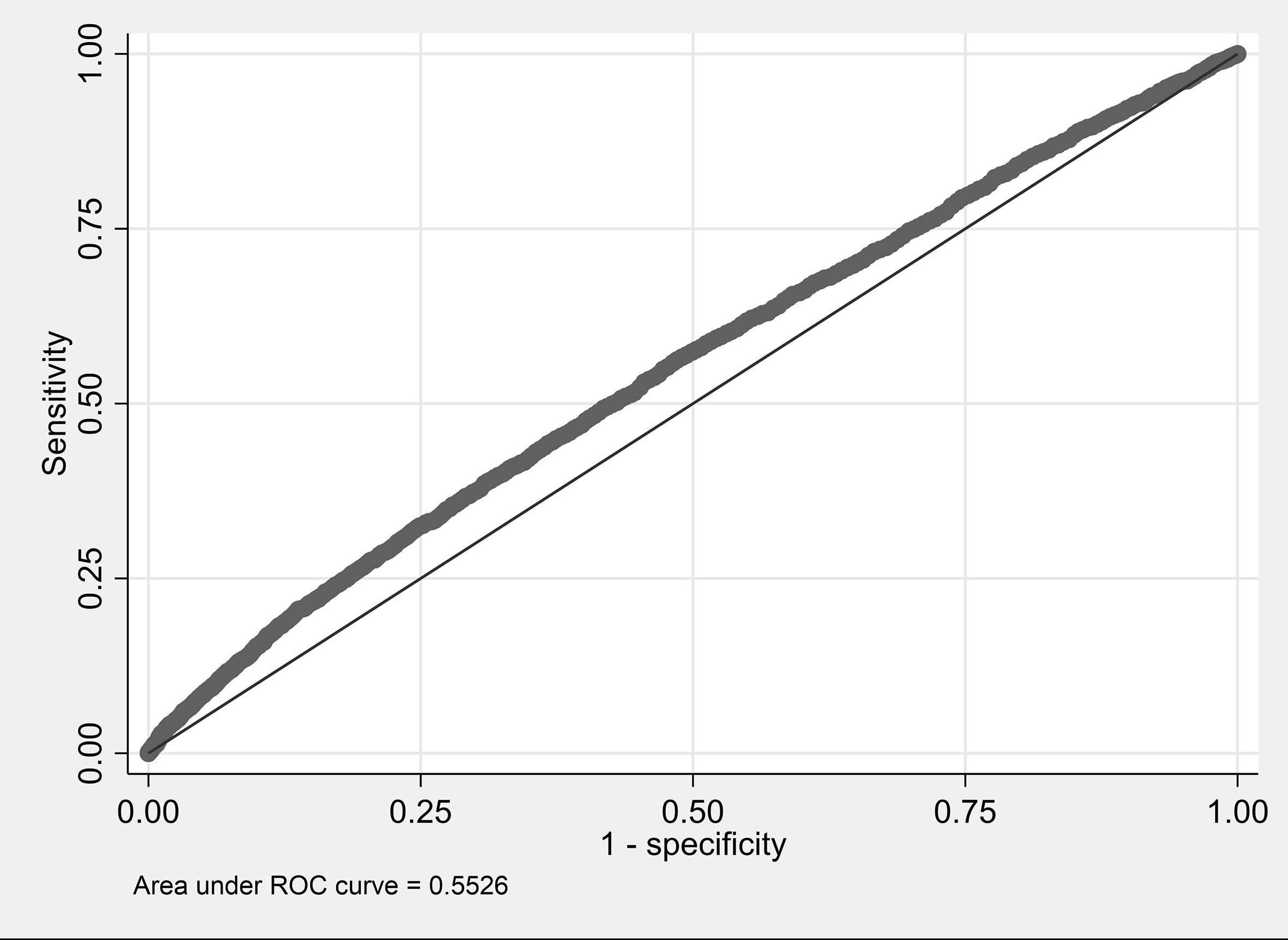

Supplement: S1 Fig — (TIF) [file pone.0255535.s004.tif]
